# VAPC, an Human Endogenous Inhibitor for Hepatitis C Virus (HCV) Infection, Is Intrinsically Unstructured but Forms a “Fuzzy Complex” with HCV NS5B

**DOI:** 10.1371/journal.pone.0040341

**Published:** 2012-07-17

**Authors:** Shaveta Goyal, Garvita Gupta, Haina Qin, Megha Haridas Upadya, Yee Joo Tan, Vincent T. K. Chow, Jianxing Song

**Affiliations:** 1 Department of Biochemistry, Yong Loo Lin School of Medicine, National University of Singapore, Singapore, Republic of Singapore; 2 Department of Biological Sciences, Faculty of Science, National University of Singapore, Singapore, Republic of Singapore; 3 Department of Microbiology, Yong Loo Lin School of Medicine, National University of Singapore, Singapore, Republic of Singapore; Centro de Biología Molecular Severo Ochoa (CSIC-UAM), Spain

## Abstract

Nearly 200 million people are infected by hepatitis C virus (HCV) worldwide. For replicating the HCV genome, the membrane-associated machinery needs to be formed by both HCV non-structural proteins (including NS5B) and human host factors such as VAPB. Recently, the 99-residue VAPC, a splicing variant of VAPB, was demonstrated to inhibit HCV replication via binding to NS5B, thus acting as an endogenous inhibitor of HCV infection. So far, the structure of VAPC remains unknown, and its interaction with NS5B has not been biophysically characterized. In this study, we conducted extensive CD and NMR investigations on VAPC which led to several striking findings: 1) although the N-terminal 70 residues are identical in VAPC and VAPB, they constitute the characteristic β-barrel MSP fold in VAPB, while VAPC is entirely unstructured in solution, only with helical-like conformations weakly populated. 2) VAPC is indeed capable of binding to NS5B, with an average dissociation constant (Kd) of ∼20 µM. Intriguingly, VAPC remains dynamic even in the complex, suggesting that the VAPC-NS5B is a “fuzzy complex”. 3) NMR mapping revealed that the major binding region for NS5B is located over the C-terminal half of VAPC, which is composed of three discrete clusters, of which only the first contains the region identical in VAPC and VAPB. The second region containing ∼12 residues appears to play a key role in binding since mutation of 4 residues within this region leads to almost complete loss of the binding activity. 4) A 14-residue mimetic, VAPC-14 containing the second region, only has a ∼3-fold reduction of the affinity. Our study not only provides critical insights into how a human factor mediates the formation of the HCV replication machinery, but also leads to design of VAPC-14 which may be further used to explore the function of VAPC and to develop anti-HCV molecules.

## Introduction

Hepatitis C virus (HCV), first discovered in 1989, infects about 200 million people worldwide [1]–[3], and is a leading risk factor for the development of severe chronic liver diseases including cirrhosis and hepatocellular carcinoma [4]. HCV is a member of the *Flaviviridae* family of enveloped, positive-stranded RNA viruses, and has a genome of approximately 9.6 kb encoding a single polyprotein of ∼3,000 amino acids, which is subsequently processed into 10 individual proteins by viral and cellular proteases [5]–[9]. Interestingly, replication of positive-stranded RNA viruses involves intracellular membrane structures, such as the endoplasmic reticulum (ER), Golgi apparatus, endosome, and lysosome [10]. The membrane-associated machinery copies the RNA genome into a negative-strand intermediate, which is then used to generate additional positive-stranded RNAs for subsequent rounds of translation and packaging into virus particles. HCV replication is initiated immediately after translation and processing of the viral protein, and all HCV gene products remain associated with intracellular membranes [11]–[17]. Although the exact mechanisms, identities of the host factors and detailed interactions among them are poorly understood, HCV nonstructural proteins including NS3, NS4A, NS4B, NS5A, and NS5B have been characterized to be the key components of the replication machinery.

**Figure 1:**
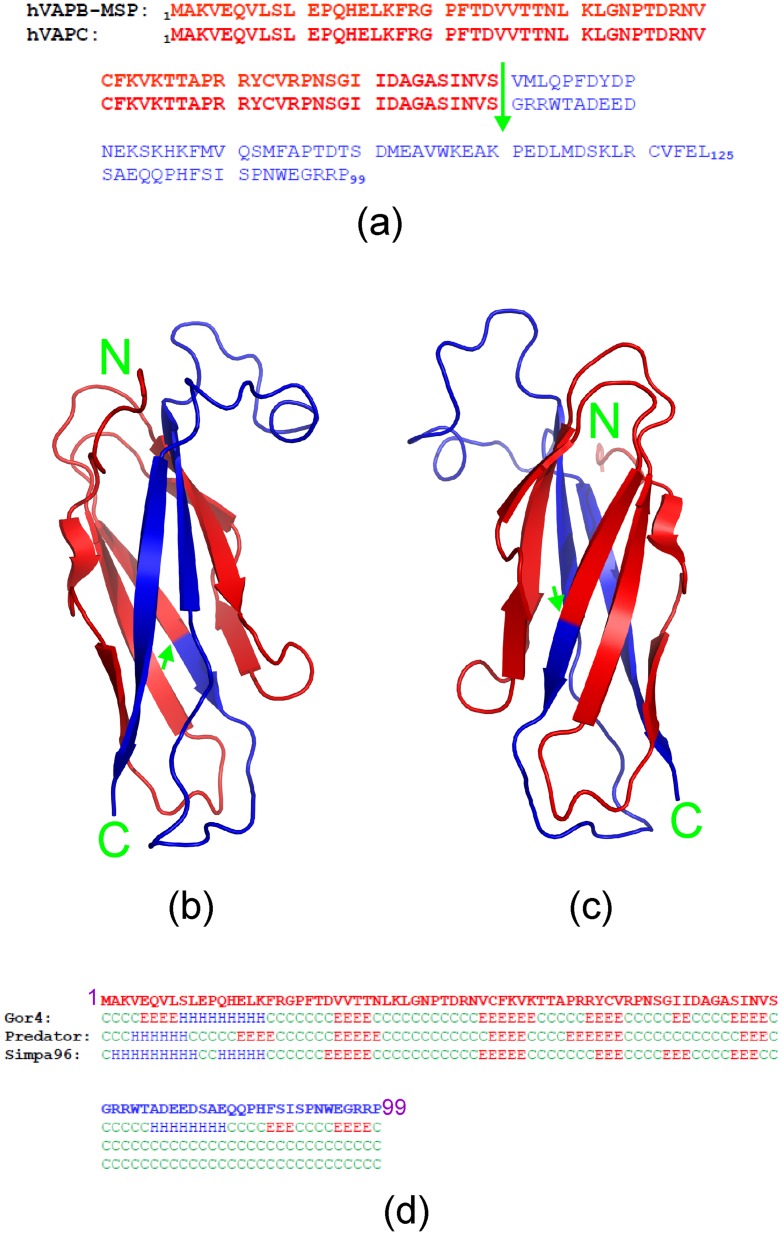
Comparison of VAPC and VAPB-MSP. (a) Sequence alignment of the human 99-residue VAPC and 125-residue VAPB-MSP domain. The first 70 residues (in red) are completely identical, while the rest (in blue) are different in the two proteins. The green arrow is used to separate the first 70 residues from the rest. (b, c) Crystal structure of the 125-residue VAPB-MSP domain that we previously determined (46), in which the 70 identical residues are in red, while the different ones are in blue. (d) Secondary-structure prediction of VAPC by computational programs GOR4, Predator, and SIMPA96. Red E, β-strand; blue H, helix; green C, random coil.

Thus far, no efficient HCV vaccine is available, and the most common therapy is based on a combination of interferon-alpha and ribavirin. However, this treatment has a success rate of only ∼50%, with severe side effects [18], [19]. As a consequence, identification of novel targets to design HCV antiviral drugs is urgently required [20]–[23]. At present, the most common targets are two viral enzymes, i.e. the NS3/4A serine protease and the NS5B RNA-dependent RNA polymerase [24], as they are amenable to the development of biochemical assays for inhibitor screening [25]. However, targeting enzymatic actions also appear to have a considerable disadvantage, i.e. rapid development of drug resistance [25], [26]. Therefore, there is promising potential to target non-enzymatic processes such as those required for RNA replication. Although how cyclophilins regulate HCV replication remains unknown, a molecular chaperone cyclophilin A that catalyzes the cis-trans isomerization of proline residues, has been demonstrated to be an important drug target for chronic hepatitis C therapy [27], [28].

**Figure 2:**
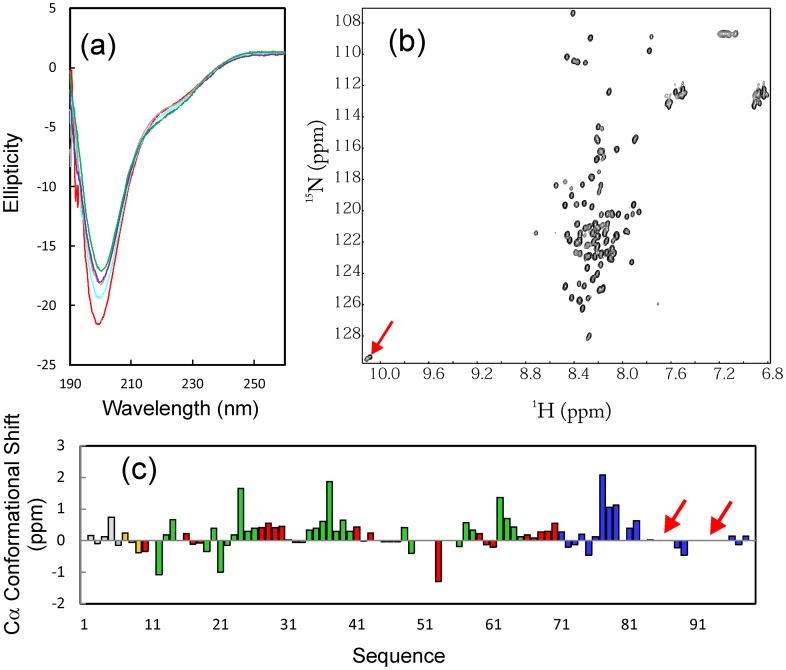
Solution conformation of VAPC. (a) Far-UV CD spectra of VAPC at different pH values, i.e. 6.5 (green); 5.5 (purple); 4.5 (grey); 3.5 (cyan); 3.0 (red). (b) ^1^H-^15^N NMR HSQC spectrum of VAPC. The HSQC peaks for two Trp side chains are indicated by the red arrow. (c) Residue-specific ^13^Cα conformational shift of VAPC derived from analysis of triple-resonance heteronuclear NMR spectra, including HNCACB and CBCA(CO)NH. Red arrows are used to indicate two regions with undetectable HSQC peaks. Blue bars indicate residues different in VAPC and VAPB-MSP domain. Residues located in the identical regions are in red if adopting β-strands; in green if adopting turn/loop conformations; in light brown if adopting helical conformation, and in grey if highly unstructured.

**Figure 3:**
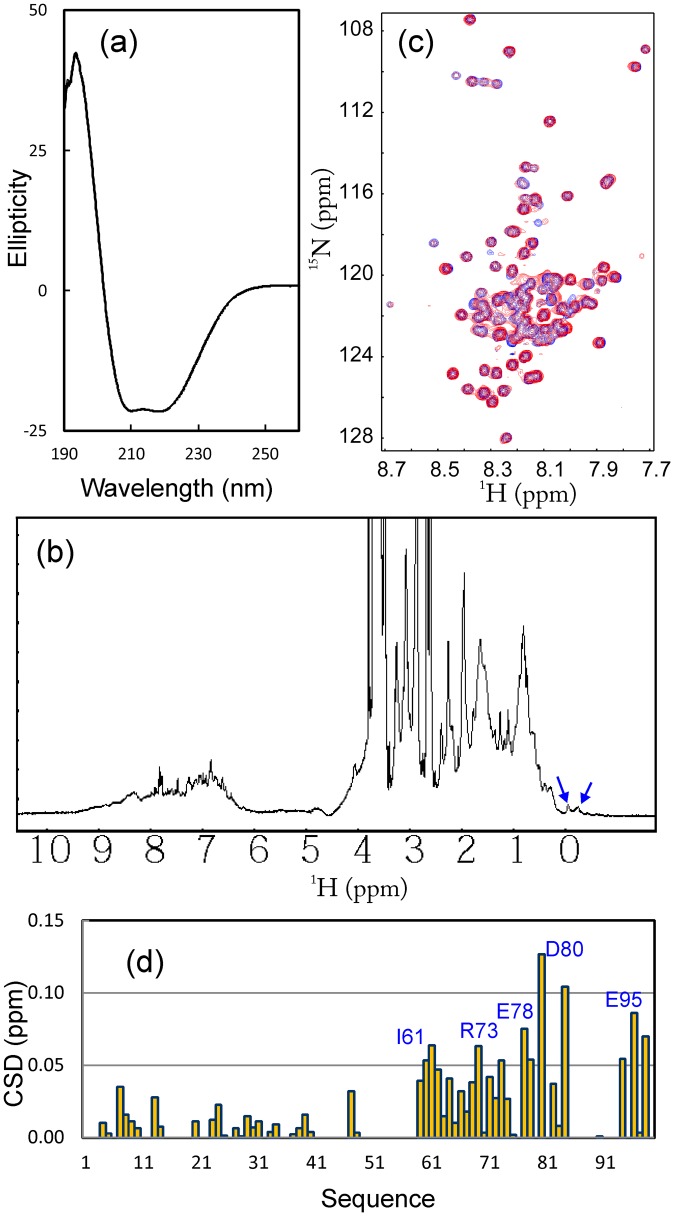
Binding between VAPC and NS5B. (a) Far-UV CD spectrum of HCV NS5B. (b) One-dimensional ^1^H NMR spectrum of HCV NS5B. Blue arrows are used to indicate very up-field NMR resonance peaks characteristic of a well-folded protein with tight tertiary packing. (c) Superimposition of ^1^H-^15^N NMR HSQC spectra of VAPC in the absence of (blue) and in the presence of unlabeled NS5B (red) at a molar ratio of 1∶1.5 (VAPC:NS5B). (d) Residue-specific changes of integrated ^1^H and ^15^N chemical shifts of VAPC in the presence of unlabeled NS5B at a molar ratio of 1∶1.5 (VAPC:NS5B).

Recently, the formation of the HCV replication machinery has been shown to require interactions of viral NS5A/NS5B to host proteins including human vesicle-associated membrane protein-associated protein (VAP) subtypes A and B [15]–[17]. NS5A is a critical component of HCV replication [29], and is additionally involved in the modulation of cell signaling pathways, interferon response, pathogenesis, and apoptosis regulation [30]. Although NS5B functions as an RNA-dependent RNA polymerase, it appears also to engage in protein-protein interactions critical for forming the replication machinery. Indeed, VAPC, a splicing variant of VAPA/VAPB, was recently identified to block the HCV RNA replication as well as HCV propagation. In particular, VAPC expression has been detected in various tissues, but negligibly in the liver. As a consequence, VAPC has been proposed to be an endogenous inhibitor of HCV infection [31], [32].

**Figure 4:**
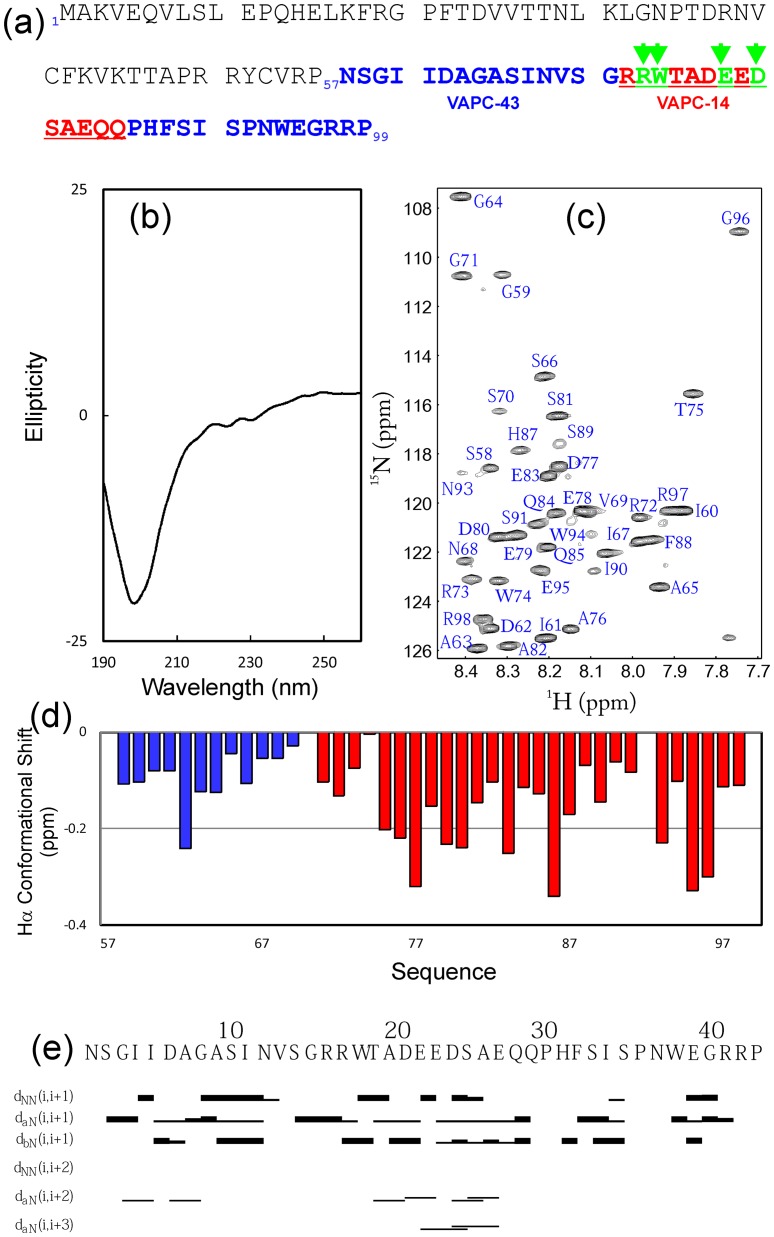
Solution conformation of VAPC-43. (a) Amino acid sequence of VAPC showing the sequences of truncated VAPC-43 (over residues 57–99) and VAPC-14 (72–85). Green arrows indicate four residues which were mutated into Ala in a VAPC-43 construct. (b) Far-UV CD spectrum of VAPC-43. (c) ^1^H-^15^N NMR HSQC spectrum of VAPC-43 with sequential assignments labeled. (d) Residue-specific Hα conformational shift of VAPC-43 derived from analysis of three-dimensional ^15^N-edited HSQC-TOCSY spectrum. Blue bars indicate residues identical to those in VAPB-MSP domain, while red ones depict VAPC-specific residues. (e) Characteristic NOE connectivities of VAPC-43 defining secondary structures derived from analysis of three-dimensional ^15^N-edited HSQC-NOESY spectrum.

The human VAP family proteins were initially identified as homologs of vesicle-associated membrane protein (VAMP)-associated protein (VAP) with a size of 33 kDa in *Aplysia californica*, including VAPA, VAPB, VAPC, and several newly-identified spliced variants [33]–[35]. VAPA and VAPB share ∼60% sequence identity, and are composed of three conserved domains, i.e. an N-terminal immunoglobulin-like β sheet domain that has 22% sequence identity to the major sperm protein (MSP); a central coiled-coil domain; and a C-terminal transmembrane domain [36]–[38]. On the other hand, the 99-residue VAPC lacking the transmembrane segment possesses the N-terminal 70 residues completely identical to the 125-residue MSP domain of VAPB, and the C-terminal 29 residues unique in VAPC (Figure 1). VAP proteins are ubiquitously expressed type II integral membrane proteins that localize to the ER and pre-Golgi intermediates [39]. Moreover, VAP proteins target lipid-binding proteins carrying a short motif containing two phenylalanines in an acidic tract (FFAT motif) to the ER [36]. The FFAT-motif consists of the consensus amino acid sequence EFFDAxE, which is conserved in several lipid-binding protein families implicated in the transfer of lipids between the ER and other organelles, such as the Golgi, endosomes, and plasma membrane [40]. The VAP proteins also interact with intracellular proteins (including Nir1, Nir2, and Nir3) via the FFAT motif which differentially affects the organization of the ER [41]. Recently, it was also shown that the VAPB-MSP domain also serves as a ligand for Eph receptors [37], [38], [42]. Strikingly, two point mutations (P56S and T46I) in the VAPB MSP domain have been identified to lead to familial amyotrophic lateral sclerosis (ALS) with rapid progression or late-onset spinal muscular atrophy [43], [44].

**Figure 5:**
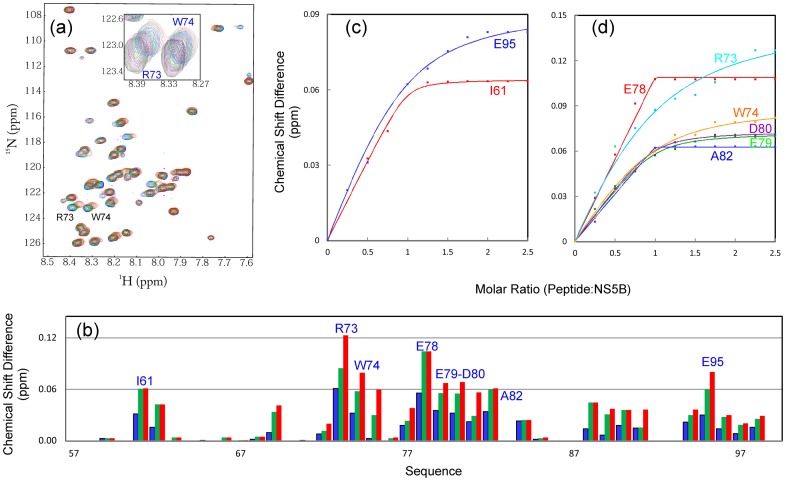
Binding of VAPC-43 with NS5B. (a) Superimposition of ^1^H-^15^N NMR HSQC spectra of VAPC-43 in the absence of (blue) and in the presence of unlabeled NS5B at molar ratios of 1∶1 (green) and 1∶2.75 (red) (VAPC-43:NS5B). The shift tracings of Arg73 and Trp74 are zoomed in, and shown as an inset. (b) Residue-specific changes of integrated ^1^H and ^15^N chemical shifts of VAPC-43 in the presence of unlabeled NS5B at molar ratios of 1∶0.5 (blue), 1∶1 (green), and 1∶2.75 (red) (VAPC-43:NS5B). (c, d) Experimental (dots) and fitted (lines) values are shown for VAPC-43 residues with significant changes of the integrated ^1^H and ^15^N chemical shifts.

Thus far, there is no structural and binding characterization of VAPC and its interaction with NS5B. Such knowledge is essential for the development of novel strategies to design inhibitory molecules against HCV infection by targeting the VAPC-NS5B interface [45]. Here, we first conducted an extensive structural characterization of the full-length VAPC by CD and NMR spectroscopy. Surprisingly, VAPC was characterized to be highly disordered in solution, only with helical-like conformations weakly populated over several segments. However, as unraveled by NMR HSQC titrations that can provide residue-specific information even for very weak bindings, VAPC was indeed capable of binding to NS5B with three discrete regions, with an average dissociation constant (Kd) of ∼20 µM. Moreover, we mapped out critical residues for binding, and subsequently obtained a 14-residue mimetic designated as VAPC-14 which only exhibited a 3-fold reduction of binding affinity to NS5B.

**Figure 6:**
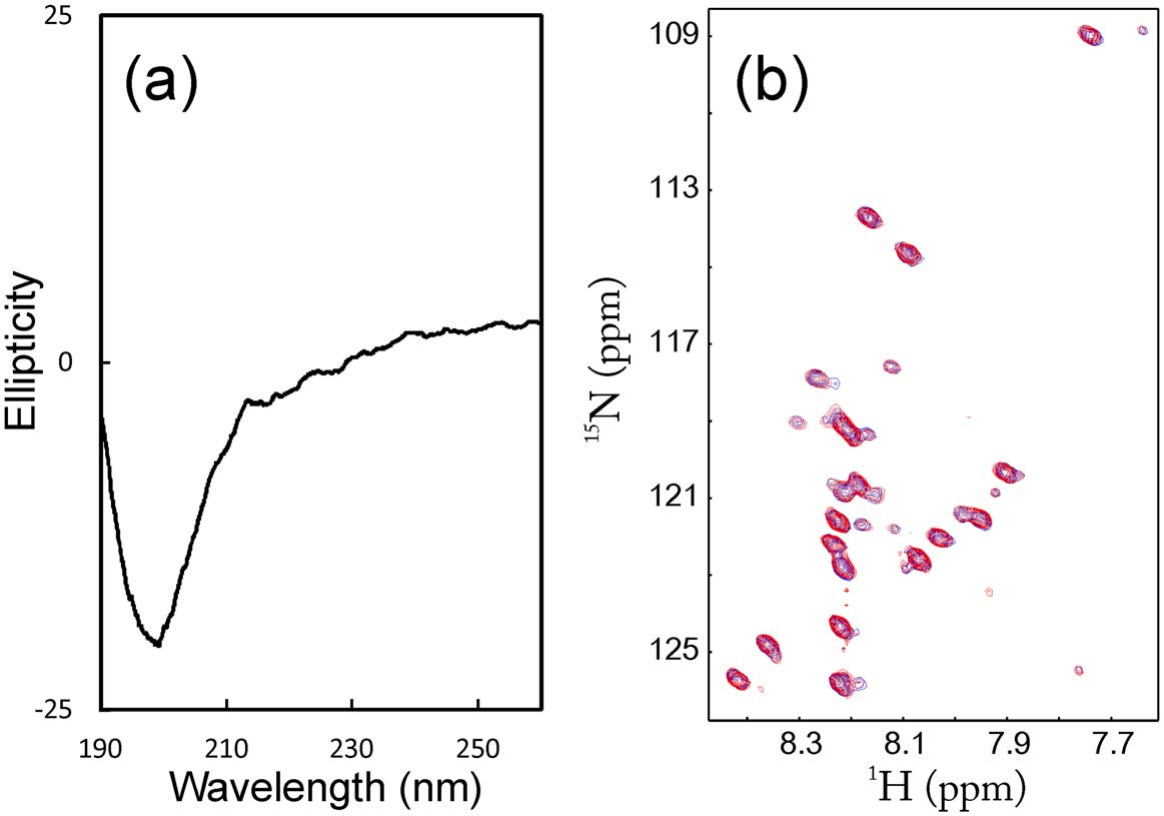
Binding of VAPC-43 mutant with NS5B. (a) Far-UV CD spectrum of VAPC-43 mutant. (b) Superimposition of ^1^H-^15^N NMR HSQC spectra of VAPC-43 mutant in the absence of (blue) and in the presence of unlabeled NS5B at a molar ratio of 1∶2.0 (red) (VAPC-43 mutant:NS5B).

## Results

### VAPC is Highly-unstructured

The recombinant VAPC protein was overexpressed as a His-tagged fusion protein in *E. coli*, and subsequently purified by Ni^2+^-affinity chromatography under native conditions. Subsequently, VAPC was separated from the His-tag by on-gel cleavage with thrombin, and was further purified by HPLC on a reverse-phase (RP) C8 column. As shown in Figure 2a, VAPC has far-UV CD spectra typical of a highly-disordered protein with pH ranging from 6.5 to 3, with the maximal negative signal at ∼198 nm, and lacking any positive signal at ∼192 nm. However, there was a small negative signal at ∼225 nm, implying that the helical conformation may be populated in VAPC to some degree. The ^1^H-^15^N NMR HSQC spectrum (Figure 2b) also indicated that VAPC lacks any tight tertiary packing as evident from its very narrow ^1^H (∼0.9 ppm) and ^15^N (∼19 ppm) spectral dispersions. Consequently, it can be concluded that VAPC is predominantly disordered, lacking of well-formed secondary structures and tight tertiary packing.

**Figure 7:**
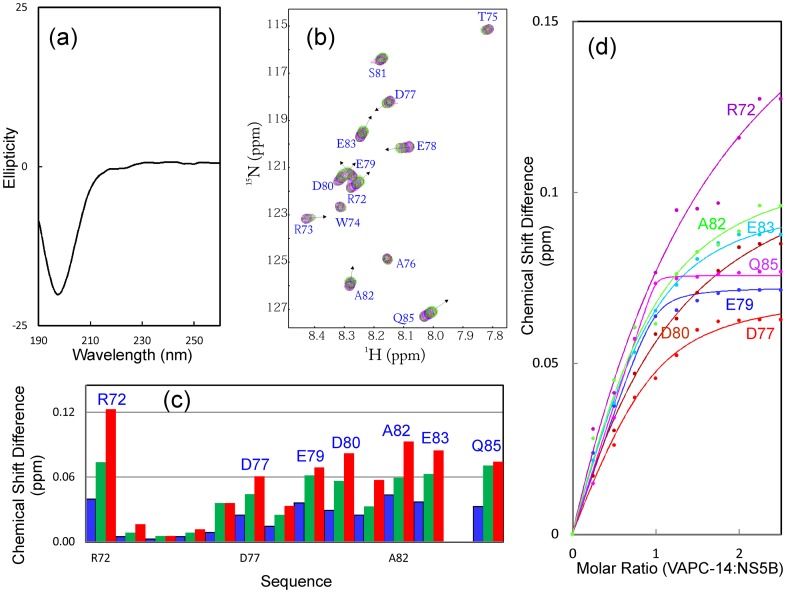
Binding of VAPC-14 with NS5B. (a) Far-UV CD spectrum of VAPC-14. (b) Superimposition of ^1^H-^15^N NMR HSQC spectra of VAPC-14 in the absence of (black) and in the presence of unlabeled NS5B at molar ratios of 0.5 (pink), 1∶1 (blue), and 1∶2.5 (green) (VAPC-14:NS5B). The sequential assignments of VAPC-14 are labeled. (c) Residue-specific changes of integrated ^1^H and ^15^N chemical shifts of VAPC-14 in the presence of unlabeled NS5B at molar ratios of 1∶0.5 (blue); 1∶1 (green) and 1∶2.5 (red) (VAPC-14:NS5B). (d) Experimental (dots) and fitted (lines) values are shown for VAPC-14 residues with significant changes of the integrated ^1^H and ^15^N chemical shifts.

By analyzing triple-resonance heteronuclear NMR spectra including HNCACB and CBCA(CO)NH acquired on a ^15^N−/^13^C-labeled VAPC sample at a protein concentration of 300 µM, we succeeded in achieving its sequential assignment and obtaining Cα conformational shifts of all residues except for several in the C-terminal portion whose HSQC peaks were not detected (Figure 2c). It is well-established that Cα chemical shift deviations from their corresponding random-coil values are very sensitive indicators of protein secondary structures, thus representing a powerful probe for detecting residual secondary structures in unfolded or partially folded proteins [46], [47]. As judged from small but positive Cα conformational shifts over the majority of VAPC residues (Figure 2c), it appeared that loop/helical-like conformations were weakly populated over several segments of the sequence, even over the first 70 residues which are identical to the VAPB-MSP domain, and adopted β-sheet secondary structures as demonstrated by our previous crystallographic and NMR characterization [37]. More precisely, over the identical 70 residues, some residues adopting β-strands in the VAPB-MSP transformed into loop or helical-like conformations in VAPC (Figure 2c).

**Table 1:** Residue-specific dissociation constants (Kd) for the binding of NS5B to VAPC-43 and VAPC-14.

Residues	VAPC43	VAPC14
	Kd (µM) ± SD	Kd (µM) ± SD
I61	1.26±19.53	
R72		161.32±63.86
R73	41.39±19.53	
W74	19.93±5.37	
D77		17.18±5.76
E78	0.01±0.07	
E79	5.52±2.06	1.41±1.34
D80	4.35±2.15	68.88±18.93
A82	0.01±0.22	27.34±12.63
E83		18.62±3.32
Q85		0.11±0.2
E95	20.27±7.63	
Average	**18.29±7.35**	**49.13±17.64**

Note: underlined Kd values have large errors, and thus were not included for calculating the average Kd values.

### VAPC Forms a “Fuzzy Complex” with NS5B

Recently, it was discovered that by interacting with HCV NS5B, the human VAPC acts as an inhibitor of HCV replication. Therefore, in the present study, we cloned and expressed the HCV NS5B protein. As shown in Figure 3a, NS5B has a far-UV CD spectrum characteristic of a well-folded helix-dominant protein, with two negative signals at ∼209 and 222 nm, as well as a very large positive signal at ∼193 nm. However, due to the short transverse relaxation time (T2) resulting from its very large molecular size, the NMR resonance peaks are very broad (Figure 3b). Nevertheless, the manifestation of several highly up-field signals clearly demonstrate that NS5B is well-folded, with a tight tertiary packing in solution.

Subsequently, we titrated the ^15^N-labeled VAPC protein with the unlabeled NS5B. As shown in Figure 3c, addition of NS5B triggered the shifts of many HSQC peaks of VAPC, indicating that VAPC was indeed able to bind to NS5B. It is also noteworthy that the HSQC spectral dispersions of VAPC did not significantly increase even upon binding to NS5B, implying that VAPC remained largely flexible even in the complex. On the other hand, based on the sequential assignment, we were able to map the perturbation of the HSQC peaks to the VAPC sequence (Figure 3d). Interestingly, the most significantly-perturbed residues are located on the C-terminal half of the VAPC sequence, mostly composed of the residues unique to VAPC (Figure 3d).

### Conformational and Binding Properties of VAPC-43

As most significantly-perturbed residues triggered by adding NS5B are located on the C-terminal half of VAPC (Figure 3d), we then cloned into a GST-fused expression vector a truncated VAPC designated as VAPC-43 spanning residues 57–99 (Figure 4a). The recombinant VAPC-43 protein was over-expressed in *E. coli*, and subsequently purified by affinity chromatography with glutathione-Sepharose 4B beads. VAPC-43 was released from the GST-fusion protein by on-gel cleavage with thrombin, and was further purified by HPLC on a reverse-phase (RP) C18 column.

As shown in Figure 4b, the 43-residue VAPC-43 is also highly disordered in solution as evident from its far-UV CD spectra. Furthermore, the lack of tight tertiary packing in VAPC-43 is clearly evident from its narrow HSQC spectral dispersions (Figure 4c). To gain residue-specific insights into the solution conformation of VAPC-43, we collected a pair of three-dimensional heteronuclear NMR spectra, namely ^15^N-edited HSQC-TOCSY and HSQC-NOESY, and subsequently achieved its sequential assignment. As judged from the negative Hα conformational shifts for most residues (Figure 4d), VAPC-43 appears to have loop or helical-like conformations weakly-populated over four segments, i.e. the first centered at Ile61-Asp62; second at Asp77-Glu78, third at Pro86, and fourth at Glu95-Gly96, similar to the patterns observed for Cα conformational shifts in the context of the full-length VAPC (Figure 3d). Interestingly, the first helical segment is composed of residues identical in both VAPC and VAPB-MSP domain. The existence of the loop or helical-like segments was further supported by characteristic NOEs (Figure 4e) over the VAPC-43 sequence defining the helical conformation, including d_NN(i, i+1)_, d_NN(i, i+2)_, d_αN(i, i+2)_, and d_αN(i, i+3)_. The existence of the loop or/and helical conformations over the VAPC-unique region is consistent with the prediction results of secondary structures (Figure 1d). However, since no d_αN(i, i+4)_ NOEs were observed, the helical conformation in VAPC-43 appears to be mainly dynamic 3_10_-helix/loop, rather than α-helix, consistent with the above CD result [56], [57].

Although it is highly unstructured in solution, it is able to bind NS5B as demonstrated by HSQC titrations of ^15^N-labeled VAPC-43 with unlabeled NS5B (Figure 5a). However, even at a molar ratio of 1∶2.5 (VAPC-43:NS5B) where the peak shifting of many residues were largely saturated (Figure 5b), the HSQC spectral dispersions still remained largely unchanged. This implies that like the full-length VAPC, VAPC-43 also remains largely flexible without any tight tertiary packing in the complex with NS5B. On the other hand, as judged from the chemical shift differences (Figure 5b), three discrete regions appears to be critical for binding with NS5B, i.e. the first one centered at Ile62, second over Arg73-Ala82, and third centered at Glu95. The first and third regions contain only ∼2 residues while the second spans ∼10 residues.

To explore the role of the second region in binding to NS5B, we thus mutated four residues (Arg73, Trp74, Glu78 and Asp80) into Ala. We successfully expressed and purified the recombinant mutant protein. The VAPC-43 mutant again appears to be highly unstructured without any well-formed secondary structures as judged from its far-UV spectrum (Figure 6a), and lacks any tight tertiary packing as demonstrated by the very narrow HSQC spectral dispersions (Figure 6b). On the other hand, the VAPC-43 mutant appears to undergo some dynamic aggregation, or/and conformational exchanges on µs to ms time-scale, and consequently the intensities of its HSQC peaks were not uniform, i.e. some were very strong but some very weak (Figure 6b). We also titrated ^15^N-labeled VAPC-43 mutant with unlabeled NS5B. Remarkably, even at a molar ratio of 1∶2.5 (VAPC-43 mutant:NS5B), no significant shift of the HSQC peaks was observed (Figure 6b), clearing demonstrating that this second region is indeed critical for binding with NS5B.

### Conformational and Binding Properties of VAPC-14

Subsequently we cloned, expressed and purified a 14-residue fragment designated as VAPC-14 containing residues Arg72-Gln85. As shown in Figure 7a, VAPC-14 is highly disordered in solution as evident from its far-UV CD spectra. Furthermore, the lack of any tight tertiary packing is also evident from its narrow HSQC spectral dispersions (Figure 7b). Nevertheless, VAPC-14 was able to bind NS5B as demonstrated by HSQC titrations of ^15^N-labeled VAPC-14 with unlabeled NS5B (Figures 7b, 7c). Again, even at a molar ratio of 1∶2.5 (VAPC-14:NS5B), the HSQC spectral dispersions still remained largely unchanged (Figure 7b). This indicates that like the full-length VAPC and VAPC-43, even in the complex with NS5B, VAPC-14 remains largely flexible without any tight tertiary packing.

We attempted to perform the ITC measurements on the binding of NS5B to VAPC and its fragments, but failed to obtain high-quality data, probably due to the complex binding mode, and/or the fact that many VAPC residues still remain largely flexible even in the complex. Therefore, to quantitatively assess the binding between VAPC-43/VAPC-14 and NS5B, we fitted the shift tracings of the VAPC-43/VAPC-14 HSQC peaks to obtain dissociation constants (Kd) as we have previously conducted on other systems [48], [49]. The fitting is exemplified in Figures 5c and d for VAPC-43 and 7d for VAPC-14, and the derived Kd values are summarized in Table 1. Strikingly, the Kd value of VAPC-14 is 49.13 µM, only ∼3-fold larger than that for VAPC-43 (18.29 µM), suggesting that deletion of the first and third binding regions only resulted in a 3-fold reduction of the binding affinity to NS5B.

## Discussion

The human VAPC is a 99-residue splicing variant of the 243-residue VAPB, with the N-terminal 70 residues being identical in both proteins. As demonstrated in the crystal structure of the human VAPB-MSP domain that we previously determined [37], the 70 residues adopt β-strand or turn secondary structures (Figure 1). Prediction of the VAPC secondary structures by several computational programs, including GOR4 [50], Predator [51], and SHIMPA96 [52], also suggest that several fragments within the identical 70 residues possess high intrinsic propensity to form β-stranded conformation (Figure 1d). Strikingly, by use of CD and NMR spectroscopy, we provide here the first residue-specific evidence that VAPC is in fact highly unstructured in solution, lacking of any stable secondary structure and tight tertiary packing. More surprisingly, NMR parameters such as conformational shifts and NOE connectivity patterns reveal that unlike the MSP domain also characterized by NMR spectroscopy [37], in VAPC, no β-strand conformation is populated. Instead, loop or helical-like conformations are identified to be weakly populated over several regions of the VAPC sequence. This observation is consistent with the notion that the formation of β-sheet conformation requires the stabilization of complex long-range interaction networks, and thus is highly context-dependent [53], [54]. Indeed, by NMR characterizations from our and other groups, the classic SH3 β-barrel fold adopted by >4,000 sequences has been previously unraveled to transform into helical conformations at certain solution conditions [55], [56] or triggered by mutations [55], [57]. In particular, we also demonstrated that one ALS-causing mutation (P56S) is sufficient to eliminate the β-barrel fold of the human VAPB-MSP domain, and to trigger the conversion into the highly-unstructured state only with weakly populated conformation [37]. Therefore, in VAPC, replacement of the remaining sequence of the 125-residue VAPB-MSP domain by 29 residues unique to VAPC is anticipated to disrupt the long-range interaction network critical for specifying or/and stabilizing the MSP fold. Consequently, VAPC would lose the ability to form β-stranded conformation, only with weakly-populated loop or helical conformations which are mostly stabilized by local interactions [53]–[57].

On the other hand, we have demonstrated that despite being highly disordered, VAPC is indeed active in binding to NS5B, with an average dissociation constant of ∼20 µM by use of NMR spectroscopy. This renders VAPC as an intrinsically unstructured protein. Interestingly, since the first 70 residues assume a well-folded β-stranded conformation in the context of the MSP domain, VAPC slightly differs from the classic “intrinsically-unstructured proteins” that usually harbor relatively degenerative sequences with a high percentage of polar and charged residues [58]–[64]. Also very surprisingly, even upon binding to NS5B, VAPC and its fragments still lack any tight tertiary packing required to manifest large spectral dispersions of the HSQC spectrum. In fact, we previously found a similar phenomenon with a transcriptional activator ApLLP for long-term memory formation, which is not only highly disordered in the free state, but intriguingly remains largely unstructured even upon forming a complex with DNA [65]. Similarly, no high-quality ITC profile could be obtained for the ApLLP-DNA interaction. Currently, this phenomenon is starting to be recognized to actually exist in a large variety of protein-protein and protein-DNA complexes involved in intrinsically unstructured proteins, and thus being designated as the “fuzzy complex” [66], [67]. The fuzzy or dynamic property of the VAPC-NS5B complex is most likely to result from its discrete multi-binding site scenario for which the binding affinity of each separate site is relatively low [68], and also many residues are not significantly engaged in binding to NS5B and consequently remain flexible (Figure 5b).

NMR characterization allows the identification of VAPC residues critical for binding to NS5B. Interestingly, it appears that three discrete VAPC regions are involved in binding with NS5B. The first centered at Ile62 and the third centered at Glu95 are very short, while the second region is much longer, spanning Arg73-Ala82 (Figure 6b). Interestingly, only the first is located within the identical sequence of VAPC and VAPB, while the second and third are both within the VAPC unique sequence. The key role of the second region was further confirmed by characterizing a VAPC-43 mutant which dramatically lost binding ability to NS5B. Nevertheless, the first and third regions also contribute to interaction with NS5B to a certain degree, since VAPC-14 (with the first and third regions deleted) exhibited a ∼3-fold loss of binding affinity (Table 1). In conclusion, VAPC-14 may be useful for exploring the biological functions of VAPC *in vivo*, and also represents a promising starting point to develop potent peptide inhibitors for interfering with the VAPC-NS5B interface in the treatment of HCV infection by use of NMR-guided approaches [68]–[70].

## Materials and Methods

### Cloning, Expression and Purification

The VAPC gene was constructed by first amplifying the DNA fragment encoding the N-terminal 70 residues identical to VAPB from our previous MSP construct [37], followed by linking to the *de novo* synthesized DNA fragment for the 29 residues unique to VAPC [48]. The genes for truncated and mutated VAPC (including VAPC-43 and its mutant VAPC-14) were generated by PCR using different pairs of primers. The full-length VAPC was cloned into pET32a expression vector, while VAPC-43 and its mutant VAPC-14 were cloned in pGEX-4T-1. The NS5B gene was PCR-amplified from the pXJ40flag-NS5B plasmid, which contains NS5B of HCV genotype 1b [71], and then cloned into pET32a.

All the expression vectors were transformed into *Escherichia coli* BL21 (DE3) Star cells (Invitrogen). For expression of recombinant proteins, cells were grown in Luria-Bertani (LB) medium in the presence of ampicillin (100 µg/ml) at 37°C to reach an absorbance of 0.6 at 600 nm, and subsequently induced with optimized IPTG concentrations. Harvested cells were resuspended and lysed by sonication in lysis buffer (50 mM Tris, 500 mM NaCl, 10% glycerol, 20 mM imidazole, 10 mM 2-mercaptoethanol, pH 7.5) containing protease inhibitor cocktail (Roche). His-tagged proteins were purified by Ni^2+^-affinity chromatography (Qiagen), while GST-fused proteins were purified by affinity chromatography with glutathione-Sepharose 4B beads (Pharmacia Biotech) under native conditions. The recombinant proteins were released from the fused tags by in-gel thrombin cleavage, and further purified either by FPLC on a Superdex 200 column for NS5B (Pharmacia Biotech), or by HPLC on RP (reverse phase) C8 and C18 columns (Vydac) for VAPC, VAPC-43 and its mutant VAPC-14, respectively. The purity of all protein samples was checked by SDS-PAGE, and their molecular masses were verified by a Voyager STR matrix-assisted laser desorption ionization time-of-flight-mass spectrometer (Applied Biosystems).

The production of the isotope-labeled VAPC and its fragment or mutant proteins for NMR studies followed a similar procedure except that the bacteria were grown in M9 medium with the addition of (^15^NH_4_)_2_SO_4_ for ^15^N labeling and (^15^NH_4_)_2_SO_4_/[^13^C]-glucose for ^15^N−/^13^C double labeling [37], [38], [48], [49]. The identities of VAPC and its fragment or mutant proteins were further confirmed by NMR assignments. The concentration of protein samples was determined by the spectroscopic method in the presence of denaturant [37], [38], [48], [49], [72].

### Circular Dichroism (CD) and NMR Experiments

All CD experiments were carried out in a Jasco J-810 spectropolarimeter (Jasco Corporation) at 25°C as previously described [37], [38], [48], [49]. The protein concentration was 20 µM in 2 mM phosphate buffer (pH 6.5) for all far-UV CD experiments.

NMR samples were prepared in 10 mM phosphate buffer in the presence of 10 mM DTT (pH 6.5). All NMR data were collected at 25°C on an 800-MHz Bruker Avance spectrometer equipped with a shielded cryoprobe as described before [37], [38], [48], [49]. For HSQC characterization, samples were prepared at a protein concentration of 100 µM. For sequential assignments of VAPC, triple-resonance NMR experiments including HNCACB and CBCA(CO)NH were acquired on a double-labeled sample at a protein concentration of ∼300 µM. For sequential assignments of VAPC-43, three-dimensional heteronuclear NMR experiments including HSQC-TOCSY and HSQC-NOESY were acquired on a ^15^N-labeled sample at a protein concentration of ∼500 µM. NMR data were processed with NMRpipe [73] and analyzed with NMRview [74].

### NMR Characterization of Binding Interactions

For NMR HSQC characterization of the binding interactions of NS5B to VAPC fragments or mutant, two-dimensional ^1^H-^15^N HSQC spectra of the ^15^N-labeled VAPC proteins were acquired at a protein concentration of 100 µM in the absence or presence of the unlabeled NS5B at different molar ratios. By superimposing the HSQC spectra at different molar ratios, the shifted or disappeared HSQC peaks could be identified, and further assigned to the corresponding residues as previously described [37], [38], [48], [49]. The degree of perturbation was reflected by an integrated chemical shift difference (CSD) calculated by the formula ((Δ^1^H)^2^+ (Δ^15^N)^2^/5)^1/2^ [48]. The CSD tracings were fitted by using the one binding site model [49] to obtain residue-specific dissociation constants (Kd) as summarized in Table 1.
